# A Cross-Sectional Audit of Body Mass Index in the Northamptonshire Early Intervention Psychosis Service

**DOI:** 10.1192/bjo.2026.11659

**Published:** 2026-06-30

**Authors:** Sanaa Moledina, Gabriela Paduret, Marlene Kelbrick

**Affiliations:** 1 Cambridgeshire and Peterborough NHS Foundation Trust, Cambridge, United Kingdom; 2 Northamptonshire Healthcare NHS Foundation Trust, Northampton, United Kingdom

## Abstract

**Aims:**

Individuals with first-episode psychosis (FEP) have a high prevalence of obesity and cardiometabolic abnormalities, compounded by antipsychotic-related metabolic side effects and unhealthy lifestyle behaviours, including tobacco smoking, physical inactivity, poor dietary habits, and substance use, resulting in an elevated risk of premature mortality. Black and minority ethnic populations exhibit elevated cardiometabolic risk, with ethnicity influencing the onset, progression, and outcomes of cardiometabolic disease. To mitigate these risks, the National Clinical Audit of Psychosis (NCAP) – Early Intervention in Psychosis standards require routine monitoring of body mass index (BMI) and intervention when thresholds are exceeded: BMI ≥25 kg/m² for the general population, compared with the lower threshold of BMI ≥23 kg/m² for South Asian and Chinese populations.

This audit aimed to establish a baseline understanding of BMI distribution within the Northamptonshire Early Psychosis Service (NSTEP), with a focus on ethnic groups at increased cardiometabolic risk, to inform future quality improvement initiatives.

**Methods:**

A retrospective audit was conducted on cross-sectional caseload data from NSTEP North and South as of 11th March 2025. BMI and ethnicity were recorded, with BMI categorized as <20 kg/m², 20–24.9 kg/m², 25–29.9 kg/m² (overweight), and ≥30 kg/m² (obese); for South Asian and Chinese patients, BMI ≥23 kg/m² was also noted. Antipsychotic prescribing patterns from a previous quality improvement project (September 2024) were included to contextualize metabolic risk.

**Results:**

Of 187 patients, 182 (97%) had BMI recorded; BMI was <20 kg/m² in 6%, 20–24.9 kg/m² in 28%, 25–29.9 kg/m² in 34%, and ≥30 kg/m² in 29%, with approximately two-thirds of the total caseload (64%) falling in the overweight or obese range, and a higher prevalence in NSTEP North than South (68% vs. 63%). Among South Asian/Asian patients, 68% (13/19) had BMI ≥23 kg/m² while Black Caribbean/African patients showed 68% (23/34) prevalence of overweight or obesity. Approximately half of patients across both services were either not prescribed antipsychotic medication or were treated with Aripiprazole, a relatively weight-neutral antipsychotic.

**Conclusion:**

This audit demonstrates that two-thirds of NSTEP patients are overweight or obese, with particularly high prevalence among South Asian/Asian and Black Caribbean/African populations, despite weight-neutral antipsychotic prescribing. These findings highlight the need for targeted, culturally informed lifestyle and weight management interventions. The plan is to introduce resource packs containing diet and exercise guidance, promote local NHS exercise and weight management programmes, encourage NSTEPactivity groups to incorporate exercise, and collaborate with the NHS Diabetes Prevention Programme to improve patient access.

